# From Rome

**Published:** 1884-02

**Authors:** J. G. Van Marter

**Affiliations:** Palazzo Marotti, 172 Via Nazionale, Rome, Italy


PALAZZO MAROTTI, 172 VIA NAZIONALE,

ROME, ITALY, December 15th, 1883.

*Editor Independent Practitioner:*

Will you allow me to call your attention, in the INDEPENDENT PRACTITIONER, to a subject which I think the members of our profession sadly neglect to mention in their discussions and writings. My attention has oftentimes been called to it while visiting my brother dentists.

CLEANLINESS.

Every surgeon knows that success in his operations means perfect cleanliness, and a proper use of antiseptics. I have had the honor, in years past, to be called to assist the late illustrious Dr. J. Marion Sims, in many operations, and I was struck with his extreme cleanliness and careful use of carbolic acid. I mention this because I believe many dental surgeons are careless or ignorant of their duty to the innocent patients intrusted to their care.

It has been my custom for a long time to cleanse every instrument I employ in carbolized water before using again, and also to wash my hands in carbolized water before touching a second patient. I fear that many an innocent person has suffered from the effects of particles of poisonous matter left on unclean instruments used in the mouths of diseased patients, who had preceded them in the dental chair.

If syphilis may be communicated by a kiss, may it not as effectually be transferred on the point of an unclean instrument?

May it not also be transferred from one patient to another, by using a mouth mirror which has not been duly carbolized? In short, do dentists as a rule exercise that carefulness in carbolizing every instrument which is essential to proper cleanliness? My observations lead me to answer, NO! I believe the use of uncarbolized instruments in probing the roots of dead teeth has often induced the very difficulty the unsuspecting dentist wished to avoid. Clean instruments, clean napkins, and clean hands, pay a large percentage on the investment, and the dividend will be cheerfully paid by appreciative ladies and gentlemen. I owe an everlasting debt of gratitude to the great Dr. J. Marion Sims, for the hints he gave me years ago on the value of thorough cleanliness, and the use of carbolized water. If others will only experience a part of the satisfaction his hints have given me, then these lines will prove to be a word to the wise.

Respectfully yours,

J. G. VAN MARTER, A. B., D. D. S.

